# MicroRNA (miRNA) in the Pathogenesis of Diabetic Retinopathy: A Narrative Review

**DOI:** 10.3390/genes16091060

**Published:** 2025-09-09

**Authors:** Stamatios Lampsas, Chrysa Agapitou, Alexandros Chatzirallis, Georgios Papavasileiou, Dimitrios Poulakis, Sofia Pegka, Panagiotis Theodossiadis, Vaia Lambadiari, Irini Chatziralli

**Affiliations:** 1 2nd Department of Ophthalmology, Attikon University Hospital, Medical School, National and Kapodistrian University of Athens, Rimini 1, Chaidari, 12462 Athens, Greece; lampsas.stam@gmail.com (S.L.); chragapitou@med.uoa.gr (C.A.); firmachat@gmail.com (A.C.); georgepapav@hotmail.com (G.P.); dpoulakis@med.uoa.gr (D.P.); sofiapegka@gmail.com (S.P.); patheo@med.uoa.gr (P.T.); 2 Diabetes Center, 2nd Department of Internal Medicine, Attikon University Hospital, Medical School, National and Kapodistrian University of Athens, Rimini 1, Chaidari, 12462 Athens, Greece; vlabadiar@med.uoa.gr

**Keywords:** diabetic retinopathy, pathogenesis, retina, miRNA, progression

## Abstract

Diabetic retinopathy (DR) is the most common microvascular complication associated with diabetes mellitus and represents a leading cause of visual impairment worldwide. Inflammation, endothelial dysfunction, angiogenesis, neurodegeneration, and oxidative stress are key pathogenic processes in the development and progression of DR. Numerous microRNAs (miRNAs) show altered expression in DR and modulate critical biological pathways. Pro-inflammatory miRNAs such as miR-155 and miR-21 promote cytokine release and vascular inflammation, while miR-146a acts as a negative regulator of Nuclear factor kappa-light-chain-enhancer of activated B cells (NF-κB) signaling. MiR-126 and miR-21 regulate endothelial integrity and angiogenesis through pathways involving Vascular Endothelial Growth Factor (VEGF). MiR-200b and miR-126 are downregulated in DR, leading to increased neovascularization via activation of the VEGF/ Mitogen-Activated Protein Kinase (MAPK) cascade. Apoptotic processes are affected by miR-195, which downregulates Sirtuin 1 (SIRT1) and B-cell lymphoma 2 (Bcl-2), promoting retinal cell death, while miR-29b downregulation permits upregulation of the transcription factor SP1, enhancing caspase-mediated apoptosis in Müller cells and endothelial cells. miRNAs collectively modulate an intricate regulatory network that contributes to the underlying mechanisms of diabetic retinopathy development and progression. This narrative review aims to summarize knowledge regarding the mechanisms miRNAs mediating pathogenetic mechanisms of DR.

## 1. Introduction

Diabetic retinopathy (DR) is the most common eye-related microvascular complication of diabetes mellitus, with a prevalence rate estimated between 30% and 40% among diabetic patients [1]. The number of people affected by DR worldwide is expected to increase markedly, from about 103 million in 2020 to 130 million in 2030, and further to 161 million by 2045, highlighting an increasing disease burden over time [2]. The pathogenesis of DR involves a complex interplay between environmental and genetic factors. However, the leading cause of DR is microvascular injury, which arises from the chronic effect of hyperglycemia due to diabetes mellitus (DM), making it the most prevalent retinal vascular disorder [3].

**Figure 1:**
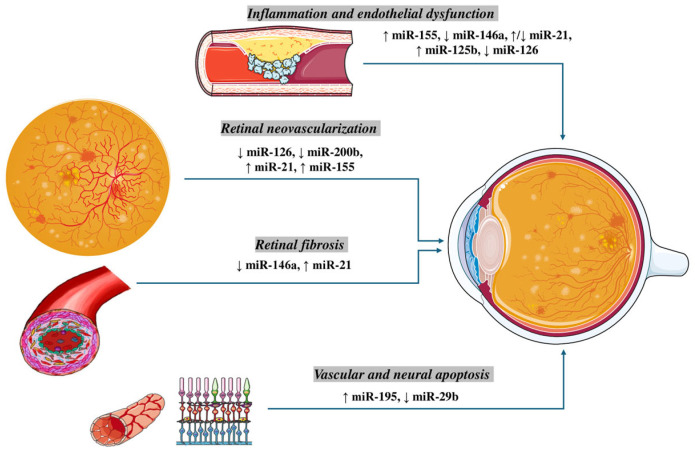
The regulation of MicroRNAs (miRs) in Pathogenesis of Diabetic Retinopathy. Inflammation and endothelial dysfunction: An upregulation of miR-155 and miR-125b, along with a downregulation of miR-146a and miR-126, have been implicated in promoting vascular inflammation, leukostasis, and impairment of endothelial cell function. Notably, miR-21 shows a context-dependent regulation, being reported as both upregulated and downregulated in different experimental settings. Retinal neovascularization: Aberrant angiogenesis in diabetic retinopathy is associated with reduced expression of the pro-angiogenic miR-126 and anti-angiogenic miR-200b, while miR-21 and miR-155 are consistently upregulated, favoring VEGF-driven neovascular responses. Retinal fibrosis: Fibrotic remodeling is driven by elevated miR-21 and decreased miR-146a, which together promote extracellular matrix deposition and activation of pro-fibrotic signaling pathways. Vascular and neural apoptosis: Increased expression of miR-195 and reduced levels of miR-29b contribute to endothelial and neuronal cell apoptosis, highlighting their role in the degenerative aspects of diabetic retinopathy.

The findings vary depending on the stage of DR, with microaneurysms, retinal hemorrhages, and vascular leakage in NPDR, and as the disease progresses to PDR neovascularization, vitreous hemorrhage, and retinal detachment are detected [4]. In the pathogenesis of DR several factors are involved, with chronic low-grade hyperglycemia-induced inflammation and oxidative stress, endothelial dysfunction, alterations in blood–retinal permeability, neovascularization playing a catalytic role by contributing to the progression of retinal microvascular damage [5,6]. Recently, the identification of microRNAs (miRNAs) has revealed their involvement in several key pathogenetic pathways contributing to the development and progression of diabetic retinopathy. MiRNAs are non-coding RNA molecules approximately 20–25 nucleotides in length that play a crucial role in the post-transcriptional regulation of gene expression, either directly by modulating gene expression or indirectly by promoting mRNA degradation [7]. Diabetic retinopathy can itself have serious complications, such as vitreous hemorrhage, retinal detachment, glaucoma, and, of course, irreversible visual impairment, making its prevention and early diagnosis very important [8]. It is diagnosed by a combination of clinical symptoms, if existing, in a patient suffering from diabetes and necessarily using Fluorescent angiography and Optical Coherence Tomography (OCT). Emerging data indicate that a wide range of miRNAs exhibit dysregulated expression in DR. With the rising prevalence of DR, this literature review aims to shed light on the role of miRNAs in the pathogenetic mechanisms that mediate disease development and progression (Figure 1).

## 2. Inflammation and Endothelial Dysfunction

Chronic low-grade inflammation is a pivotal contributor to the pathogenesis and progression of diabetic retinopathy. Persistent hyperglycemia fosters a detrimental microvascular metabolic environment that activates multiple inflammatory pathways [9]. In this context, a wide range of inflammatory mediators—such as adhesion molecules, cytokines, chemokines, and regulatory factors—are upregulated in the diabetic retinal microvasculature [10,11,12]. Hyperglycemia also promotes oxidative stress through excessive production of reactive oxygen species (ROS), activation of inflammasomes, and induction of a pro-inflammatory phenotype [13]. Importantly, microRNAs (miRNAs) have emerged as key post-transcriptional regulators within these inflammatory cascades.

### 2.1. MiR-155 and Inflammation

MiR-155 consists of RNA duplexes of ~22 nucleotides and is encoded by a gene that is activated in B-cell lymphomas and various inflammatory diseases [14], called the B-cell Integration Cluster (BIC) gene [15]. MiR-155 upregulation is associated with the macrophage polarization toward the “M1” pro-inflammatory phenotype instead of their “M2” anti-inflammatory phenotype, enhancing the production of pro-inflammatory cytokines such as IL-6 and TNF-α, which are central mediators of diabetic tissue injury [16,17]. Moreover, miR-155 upregulation enhances the negative feedback inhibitor of JAK/STAT signaling, resulting in a hyperactive JAK/STAT pathway that progresses to an upregulated expression of pro-inflammatory cytokines such as IL-6 and IL-12 [17]. Particularly, these cytokines contribute significantly to the breakdown of blood–retinal barriers, affecting the tight junction proteins and inducing apoptosis of retinal endothelial cells and pericytes [18]. Additionally, miR-155 has a pivotal role in the prolonged atherosclerotic plaque inflammatory process since it promotes further oxidation of Low-Density Lipoprotein (LDL) in oxidized LDL (oxLDL), endothelial Nitric Oxide (NO) release, and adhesion molecule expression by progressing leukocyte adhesion and transmigration, which affects the whole retinal microvasculature [19,20]. Furthermore, miR-155 demonstrates a regulatory role on vascular smooth muscle cells (VSMCs) proliferation and neointima formation, due to its contribution to inflammatory plaque progression and stability [21,22]. In particular, in a case–control study of 170 patients with and without DR, miR-155 expression levels higher in DR patients [23]. In another case–control study of 80 subjects, expression levels of miR-155 were associated with DR severity, since its levels were higher in patients with proliferative retinopathy compared to patients with non-proliferative retinopathy [24].

### 2.2. MiR-146a and Inflammation

MiR-146a has emerged as an important regulator in the pathogenesis of DM and its vascular complications, including DR [25]. MicroRNA-146a, encoded by the MIR146A gene, represents a small non-coding RNA molecule of ~22 nucleotide duplexes that exerts its function by targeting critical mediators of inflammation such as IRAK1, the tumor necrosis factor (TNF) transcript, and IL-1β [26]. Functionally, miR-146a has a key protective negative regulation in maintaining immune system balance and regulating both innate and adaptive immune responses, since it dampens the activity of the nuclear factor kappa-light-chain-enhancer of activated B cells (NF-κB) signaling pathway [27]. NF-κB is a key transcription factor that drives inflammation by inducing the expression of pro-inflammatory cytokines, chemokines, and immune regulators [28]. Particularly, miR-146a acts downstream of toll-like receptors (TLRs), leading to the activation of interleukin-1 receptor-associated kinase 1 (IRAK1), tumor necrosis factor receptor-associated factor 6 (TRAF6) [29]. In the absence of miR-146a, macrophages exhibit heightened responses, with increased TNF-α, interleukin 6 (IL-6), and interleukin 12 (IL-12) levels [30]. In diabetic patients, hyperglycemia activates NF-κB, resulting in an increase in ROS production via upregulation of NADPH oxidase (NOX) enzymes and pro-inflammatory cytokines [31]. By targeting TRAF6 and IRAK1, and inhibiting NF-κB activation, miR-146a leads to decreased mitochondrial ROS generation and reduced cytokine-induced oxidative burst, resulting in a suppression of oxidative stress [32]. Hence, miR-146a is a key negative regulator of inflammation, often considered a homeostatic or compensatory “brake” on retinal pro-inflammatory signaling. Feng et al. first demonstrated that miR-146a, enriched in retinal endothelial cells, is reduced in diabetic conditions, and that intravenous delivery of miR-146a restores its retinal endothelial levels [33]. Interestingly, in retinal tissue of diabetic rats, miR-146a showed a negative correlation with NF-κB, TNF-α and HOMA-IR (insulin resistance), and a positive correlation with Nuclear factor erythroid 2-related factor 2 (Nrf2)—an antioxidant regulator- which demonstrated that its downregulation in diabetic conditions correlates with increased inflammation, oxidative stress, and insulin resistance [34].

### 2.3. MiR-21 and Inflammation

MiR-21 is one of the most widely studied inflammatory miRNAs and plays a complex immunomodulatory role in diabetic complications, including DR, being upregulated [35]. It regulates gene expression at the post-transcriptional level and modulates both the NF-κB and NLRP3 inflammasome pathways, which are activated by pathogen- and damage-associated molecular patterns (PAMPs and DAMPs) [36,37]. Moreover, by targeting tumor suppressor genes miR-21 inhibits NF-κB activation and lowers IL-6 levels while upregulating IL-10, it contributes to an overall anti-inflammatory response [38]. Notably, another study in humans showed that miR-21 was positively correlated with type 2 diabetes (T2D), HbA1c and HOMA-IR, showing also diagnostic value for detecting and assessing the severity of DR [39]. However, miR-21′s role in inflammation is complex since it acts either as a suppressor or promoter of inflammation. In pro-inflammatory pathways, miR-21 promotes in pro-inflammatory pathways in certain cells by TNF/IFNγ production in T-cells [38]. A large case–control study of 90 PDR cases, 90 matched NPDR patients revealed that miR-21 expression levels were significantly elevated in severe DR stages [40]. Given this multifaced inflammatory regulation of miR-21, a similar immunomodulatory role has been shown miR-125b. Particularly, miR-125 has an anti-inflammatory role by suppressing the monocyte chemotactic protein-1 (MCP-1) expression, that involved monocyte recruitment and vascular inflammation [41]. A recent case–control study reported that in the vitreous exosomes of patients with PDR miR-125 was significantly dysregulated miRNA [42]. Moreover, due to miR-125 high expression in retinal pigment epithelium (RPE) cells, high-glucose conditions in patients with diabetic retinopathy induce the epithelial–mesenchymal transition (EMT) of RPE cells, which is believed to play a critical role in the onset of fibroproliferative diseases like PDR [43].

### 2.4. MiR-126 and Inflammation

MiR-126 is an endothelial-enriched miRNA that plays a crucial role in preserving vascular integrity, making it highly relevant to the pathogenesis of DR. The retinal microvasculature depends on intact endothelial function for maintenance of the blood–retinal barrier, vascular tone, hemostasis, leukocyte adhesion, and angiogenesis [6]. Under hyperglycemic conditions, miR-126 regulates multiple pathogenetic pathways that otherwise contribute to endothelial injury and BRB disruption [44]. In high glucose-treated human retinal capillary endothelial cells miR-126 was shown to reduce experimental diabetic retinopathy and suppress endothelial cell proliferation by targeting polo-like kinase 4 (PLK4), which is a serine/threonine kinase playing a crucial role in cell cycle regulation, whose overexpression can lead to aberrant proliferation and apoptosis of endothelial cells [45]. Moreover, in a large observational study, serum miR-126 showed high sensitivity and specificity in discriminating NDR and NPDR from healthy controls, being an important biomarker for screening retinal endothelial injury [46]. Moreover, in pathological hyperglycemic conditions, the transfer of active miR-126 facilitates the repair of damaged vascular endothelium via endothelial-derived microparticles [47]. In addition, miR-126 in conditions of vascular stress, such as hyperglycemia, showed to maintain endothelial function and barrier integrity by downregulating TGFβ expression that is involved in the regulation of endothelial apoptosis and permeability [48]. In patients with DR, overexpression of miR-126 was shown to reduce ROS generation and apoptosis in microvascular endothelial cells [49]. In in vitro model demonstrates a protective role of miR-126 in the endothelium, since its downregulation contributes to endothelial dysfunction by reducing HIF-1α (hypoxia-inducible factor 1-alpha), a factor involved in vascular repair [47].

### 2.5. MiR-21 and Endothelial Dysfunction

MiR-21 also plays a pivotal role in the regulation of endothelial function, which is central to the pathogenesis of diabetic retinopathy (DR). In diabetic mice, overexpression of miR-21 suppresses peroxisome proliferator–activated receptor-α (PPARα), a key regulator of lipid metabolism and inflammation that exerts protective effects in retinal endothelial cells. This downregulation attenuates the therapeutic benefits of PPARα signaling, including vascular repair, inflammatory modulation, and protection against apoptosis [50]. Furthermore, in a human study in conditions of high glucose and insulin in the blood, miR-21 expression resulted in upregulation of ET-1 and downregulation of NO secretion, leading to endothelial cell dysfunction [51]. MiR-21 also enhances endothelial activation by increasing the expression of vascular cell adhesion molecule-1 (VCAM-1) and monocyte chemoattractant protein-1 (MCP-1), thereby promoting leukocyte adhesion and transmigration—processes that are pivotal for chronic retinal microvascular inflammation [52]. MiR-21 targets several autophagy-related genes and modulates the autophagy pathway across various diseases [53]. It is thought to contribute to the post-translational regulation of autophagy, with miR-21 being among the most extensively investigated miRNAs. In diabetic mice, retinal and endothelial cell miR-21 levels increase via NF-κB activation. MiR-21 protects ECs from high-glucose–induced apoptosis by suppressing death domain-associated protein, with inhibition of miR-21 worsening glucose toxicity [54]. Both miR-204-5p and miR-1273g-3p are increased in diabetic retinopathy. MiR-204-5p worsens the disease by blocking autophagy in retinal cells, while miR-1273g-3p promotes disease progression by affecting the autophagy-lysosome pathway [55,56] (Table 1).

## 3. Retinal Neovascularization

Retinal neovascularization (NV), the formation of abnormal new blood vessels in the retina, represents a defining feature of PDR and is a major cause of severe and irreversible vision loss [57,58]. Complications arising from NV, such as vitreous hemorrhage and tractional retinal detachment, frequently lead to profound visual impairment in affected patients [57]. The process of NV reflects a complex imbalance between pro- and anti-angiogenic factors, which govern endothelial cell proliferation, migration, and maturation [59]. Key angiogenic drivers in the diabetic retina include vascular endothelial growth factor (VEGF), insulin/insulin-like growth factors (IGF), and hypoxia-inducible factor-1 alpha (HIF-1α), which are upregulated in response to hyperglycemia and retinal ischemia [59,60,61,62,63]. At the post-transcriptional regulation level of those angiogenic stimulators, miRNAs are considered as one of the most important regulators [64]. Beyond VEGF-dependent mechanisms, intracellular signaling cascades such as p38 MAPK, ERK, protein kinase C (PKC), and STAT3 contribute to the proliferative and migratory phenotype of retinal endothelial cells under ischemic stress [65,66,67]. Furthermore, v-ets erythroblastosis virus E26 oncogene homolog 1 (Ets-1) is a transcription factor that regulates genes involved in angiogenesis, with Matrix Metalloproteinase-1 (MMP-1) and Vascular Endothelial Growth Factor Receptor 2 (VEGFR2) being key Ets-1-associated mediators for extracellular matrix remodeling and endothelial migration. [68]. During angiogenesis, Matrix Metalloproteinases (MMPs) are the proteins that play a major role in migration of endothelial cells, as the initial steps of the endothelial cells surrounding extracellular matrix degradation is induced by them [69]. Additionally, the transcriptional co-activator p300, through its histone acetyltransferase activity, mediates glucose-induced activation of transcription factors and upregulation of vasoactive mediators, including VEGF and endothelin-1 (ET-1), thereby amplifying the pro-angiogenic response [70]. Collectively, these findings underscore that retinal NV in DR is driven by the interplay of hypoxia-inducible growth factors, signaling cascades, transcriptional regulators, and extracellular matrix–remodeling enzymes. Understanding how miRNAs intersect with these angiogenic pathways provides a promising avenue for developing targeted therapies to suppress pathological NV while preserving physiologic vascular repair.

### 3.1. MiR-126 and Neovascularization

MiR-126 is an endothelial-specific miRNA transcribed from the seventh intron of the endothelial growth factor-like 7 (EGFL7) gene on chromosome 9 and is a critical regulator of vascular development and angiogenesis [71,72]. During physiological vascular growth, miR-126 modulates the VEGF/PI3K/MAPK signaling axis, thereby controlling endothelial proliferation and survival [71,73]. Knockout models demonstrate their essential role, as loss of miR-126 results in severe embryonic vascular dysplasia, intracranial hemorrhage, and vascular rupture [74,75]. MiR-126 exerts its inhibitory effect on VEGF expression by targeting a site in the 3′ untranslated region of its mRNA and thus, the expression of VEGF is upregulated [72,76,77]. Furthermore, according to the experimental study of Yanyan Bai et al., using OIR (oxygen-induced retinopathy) mice, proved that miR-126 regulates the angiogenic growth factors through p38 and ERK MAPK pathway. As the production of the angiogenic factors VEGF, IGF-2, and HIF-1α—which are regulated by miR-126—may depend on the p38 and ERK enzymes of the MAPK pathway, it is likely that in ischemic retina, reduction in miR-126 level stimulates the activation of p38 and ERK, which increase the expression of the downstream angiogenic factors. Moreover, the above team proposed as an extra mechanism of the VEGF and IGF-2 upregulation; miR-126 targets a base pairing site in the 3′- untranslated region (3′-UTR) of the VEGF and IGF-2 mRNAs, inhibiting their translation [78]. Additionally, miR-126 may stop hypoxia-induced retinal neovascularization by suspending the cycle progression of the retinal endothelial cells and inhibiting MMP-9 expression [77]. Panpan Ye et al., in their experimental study in hypoxia-treated RF/6A cells and STZ-DM rats diabetic retinas showed that under hypoxic conditions found in diabetic retinas, MiR-126 expression is downregulated [77].

### 3.2. MiR-200b and Neovascularization

MiR-200b is a member of the miR-200 family, it is transcribed from chromosome 1 [79], and it is expressed in multiple cell types, including cancer cells, stem cells, and endothelial cells [68,80,81]. It upregulates key cellular processes such as migration, proliferation, and apoptosis [80]. Functionally, miR-200b acts as an anti-angiogenic factor, and its reduced expression has been shown to promote endothelial angiogenesis [82]. In the context of diabetic retinopathy, miR-200b expression is consistently downregulated in human diabetic retinas. Mechanistically, Chan et al. demonstrated that miR-200b directly interacts with the 3′UTR of Ets-1 mRNA in human dermal microvascular endothelial cells (HMECs), inhibiting its translation. Under hypoxic conditions, however, miR-200b is downregulated, which derepresses Ets-1 expression, thereby inducing MMP-1 and VEGFR2 expression and promoting angiogenesis [68]. Li EH et al. in their case–control study with 255 Diabetic Retinopathy patients and 253 healthy people, confirmed that in diabetic retinas miR-200b is significantly downregulated, whereas VEGFA is significantly upregulated, while they confirmed that miR-200b can form partial base pairs with the 3′ UTR region of the VEGFA mRNA and inhibit its expression [83]. Furthermore, McArthur K et al. using retinal tissue from streptozotocin-induced diabetic rats and glucose-exposed endothelial cells from Human Umbilical Vein in their experimental study, showed that hyperglycemia in diabetes induces downregulation of miR-200b in rat diabetic retinas, which causes VEGF mRNA upregulation [82]. They also confirmed that miR-200b exerts its inhibitory effect on VEGF expression by targeting a seed sequence in the 3′ untranslated (UTR) region of the VEGF mRNA, and thus, the expression of VEGF is upregulated in diabetic retinas [82]. In the same research study, it is confirmed that hyperglycemia induced miR-200b downregulation, resulting in increased expression of the transcriptional coactivator p300, through which the levels of vasoactive factors Endothelin-1 (ET-1) and VEGF are increased [82].

### 3.3. MiR-21 and Neovascularization

MiR-21 is located on 17q23-2 chromosome and has a key role in cell viability, angiogenesis, and inhibition of apoptosis, and is also linked to various physiological and pathophysiological processes, including angiogenesis, glucose regulation, and the development of diabetes and its associated microvascular and macrovascular complications [35,84,85]. Experimental evidence strongly supports its pro-angiogenic role in diabetic retinopathy. In streptozotocin (STZ)-induced DR rat models, Jian-Min Lu et al. demonstrated that miR-21 promotes angiogenesis through the PTEN/PI3K/Akt pathway. Specifically, miR-21 binds to the 3′UTR of PTEN mRNA, suppressing its translation, leading to PTEN downregulation [86]. In the retinal tissue of DR rats, expression of miR-21 is upregulated, and thus, PTEN level expression is downregulated [86], leading to the concomitant upregulated expression of the PI3K/Akt signaling downstream cascade [86,87]. While the typical insulin signaling pathway involves the IRS-1/PI3K/Akt cascade and its downstream molecules, which trigger the transcription of VEGF mRNA and the expression of VEGF, miR-21-induced PTEN upregulation promotes retinal neovascularization through the activation of PI3K/Akt/VEGF signaling pathway [86,87,88,89,90]. Complementary in vitro findings from Feng Qiu et al. using human retinal microvascular endothelial cells (HRMECs) exposed to high glucose showed that miR-21-5p expression is significantly upregulated under hyperglycemic conditions. Elevated miR-21-5p suppresses maspin, an anti-angiogenic protein, thereby enhancing PI3K/Akt and ERK pathway activation [91]. Under high glucose conditions, miR-21-5p expression is upregulated, causing maspin downregulation, which promotes the activation of PI3K/ATK and ERK pathways, inducing angiogenesis. Also, increased VEGF and VEGFR2 mRNA and protein, related to miR-21-5p upregulation levels, are found in high glucose-treated HRMECs, promoting angiogenesis [91]. Taken together, these findings indicate that upregulated miR-21 in DR directly promotes angiogenesis by targeting negative regulators such as PTEN and maspin, resulting in activation of PI3K/Akt and ERK signaling, VEGF upregulation, and enhanced endothelial proliferation. Thus, miR-21 represents a crucial pro-angiogenic driver in the diabetic retina and a potential therapeutic target for controlling pathological neovascularization.

### 3.4. MiR-155 and Neovascularization

MiR-155, located on human chromosome 21, is involved in various biological functions, such as microglia stimulation, lymphocyte activation, and immune cell regulation [91,92,93]. Zhuang Z et al., in their experimental study using VEGF-treated HRMECs, OIR mice, and laser photocoagulation-induced choroidal neovascularization (CNV) mice, investigated the role of miR-155 in retinal neovascularization [94]. MiR-155 exerts its inhibitory effect on SHIP1 expression by targeting a site in the 3′ untranslated region of its mRNA [95,96]. The research group confirmed that in the retinal neovascularization animal model, miR-155 is upregulated, downregulating SH2-containing inositol 5′-phosphatase 1 (SHIP1), and consequently promoting PI3K/Akt activation, and particularly p-Akt (Ser 473), inducing angiogenesis [94]. These findings suggest that miR-155 acts as a pro-angiogenic regulator in retinal neovascularization by repressing SHIP1 and enhancing PI3K/Akt signaling. Its dual role in both immune modulation and angiogenic regulation highlights miR-155 as a potential therapeutic target for controlling pathological neovascularization in diabetic retinopathy.

## 4. Fibrosis

PDR is the leading cause of blindness, primarily due to tractional retinal detachment resulting from fibroproliferative responses driven by elevated intraocular levels of biologically active growth factors [97]. Fibrosis, characterized by the disordered accumulation of extracellular matrix (ECM) proteins and fibers, disrupts tissue architecture and cellular function. In DR, hyperglycemia-induced fibrosis contributes to retinal vascular damage, tissue disorganization, and vision impairment [98,99,100]. Among the ECM proteins, fibronectin (FN) is upregulated through hyperglycemia-induced abnormal signaling [99,101,102]. Furthermore, as part of the pathogenesis of diabetic retinopathy, high glucose levels promote Endothelial to Mesenchymal Transition (EndMT), leading to fibrosis progression [103]. TGF-β, like in other diabetes-related disorders, plays a crucial role in mediating EndMT mainly through SMAD signaling activation [103,104]. Collectively, these mechanisms highlight how hyperglycemia drives ECM accumulation and cellular transdifferentiation, establishing a fibrotic microenvironment that underpins PDR development and retinal structural deterioration.

### 4.1. MiR-146a and Fibrosis

MiR-146a is a key regulator of the innate immune response and is involved in various inflammatory processes [105,106,107,108]. As miR-126a exerts its inhibitory effect on FN expression by targeting a site in the 3′ UTR of the FN mRNA, the research group confirmed, glucose-induced miR-146a downregulation in high glucose-treated HUVECs and retinas of STZ-induced diabetic rats, upregulates FN expression [33]. Thus miR-146a miR-146a-dependent FN upregulation as an ECM protein, accumulates in the retinal tissue, leading to structural alterations such as basement-membrane thickening, mesangial matrix expansion and focal scarring [99,101]. Feng B et al. in their experimental study using high glucose treated HUVECs and STZ-induced diabetic male SD rats investigated if miR-146a expression affects FN production in the diabetic retina [33]. Under high glucose conditions in diabetic retinas, miR-146a is downregulated in the endothelial cells [33].

### 4.2. MiR-21 and Fibrosis

Other than miR-21′s role in angiogenesis, it also plays a vital role in fibrosis. Usui-Ouchi A et al. in their case–control study recognized miR-21 as a potential disease-modifying miRNA in the vitreous humor associated with the development of retinal fibrosis [109]. According to the study miR-21 expression in Retinal Pigment Epithelial Cells (RPECs) is upregulated due to high glucose and TGF-β2, both of which are relevant to diabetes. MiR-21 overexpression promotes proliferation and migration of RPECs, inducing fibrosis [109]. Cao Y et al. in their experimental study using HRMECs, STZ-induced diabetic mice and Transgenic mice, showed that hyperglycemia induces EndMT of ECs in diabetic retina via TGF-β upregulation which is regulated by MiR-200b downregulation [103].

## 5. Apoptosis

Apoptosis is the process of programmed cell death [110]. Diabetic retinopathy is considered to be both vascular and neural disease [110,111,112], as various neuronal abnormalities and neuronal apoptosis have been reported prior to retinal vascular system abnormalities, causing disturbances in color vision, dark adaptation, and electrophysiological measures [112,113,114]. Müller cells, being the main neuroglial cells in the retina, are crucial for sustaining normal retinal function, while their apoptosis happens before the microvascular alterations of the diabetic retina [111,112,113,114,115]. Furthermore, Silent Information Regulator protein SIRT1, as a NAD+—dependent deacetylase, has a significant role in metabolic regulation and adaptation and is extensively involved in regulating inflammation, oxidative stress, autophagy, and apoptosis [116]. Downregulation of SIRT1 has an important role in DR, since reduced activity leads to hyperacetylation of transcription factors linked to oxidative stress, inflammation, mitochondrial dysfunction, and apoptosis in the Diabetic Retina [117].

### 5.1. MiR-195 and Apoptosis

MiR-195 located on the 17p13.1 chromosome is expressed in various diseases including diabetic retinopathy [118,119]. MiR-195 inhibits cell proliferation and induces apoptosis in various types of cancer and cardiovascular diseases [118,119,120,121,122,123]. Shan et al. in their case–control study (50 patients with DR undergoing vitrectomy as the cases and 40 patients with idiopathic macular holes undergoing vitrectomy as the controls) with additional cell-based experiments using high glucose treated HRECs and HMECs, associate hyperglycemia induced miR-195 upregulation with SIRT1 reduction and concomitant acceleration of cell apoptosis in the diabetic retina [124]. In the retinal tissue of DR patients, miR-195 upregulation downregulates SIRT1 expression promoting apoptosis. As SIRT1 can decrease the ratio of the proapoptotic protein BAX to the antiapoptotic protein BCL-2, thereby preventing apoptosis [125,126], in the DR retinal tissue, SIRT1 regulated proapoptotic BAX is found increased, while SIRT1 regulated antiapoptotic BCL-2 is found decreased, thereby promoting miR-195-induced apoptosis [124]. Mortuza R et al. using High-glucose treated Human dermal microvascular Endothelial Cells (HMECs) and Human retinal microvascular Endothelial Cells (HRECs) and retinal tissue from STZ-induced diabetic rats in their experimental study, investigated the regulatory role of miR-195 in microvascular changes associated with hyperglycaemia in diabetic retinopathy [119]. The research team confirmed the regulatory role of miR-195 in SIRT1 expression in diabetic retinopathy [119]. According to the team, miR-195 exerts its inhibitory effect on SIRT1 expression by binding to the 3′ UTR of SIRT1 mRNA, thus inhibiting its translation [119]. Hyperglycemia induces miR-195 upregulation and consequently downregulation of SIRT1 expression in the ECs of diabetic retinas, and thus SIRT1-regulated antioxidant MnSOD downregulation accelerating aging-like changes in the vascular ECs and promoting cellular senescence in the retina [127].

### 5.2. MiR-29b and Apoptosis

**Figure 2:**
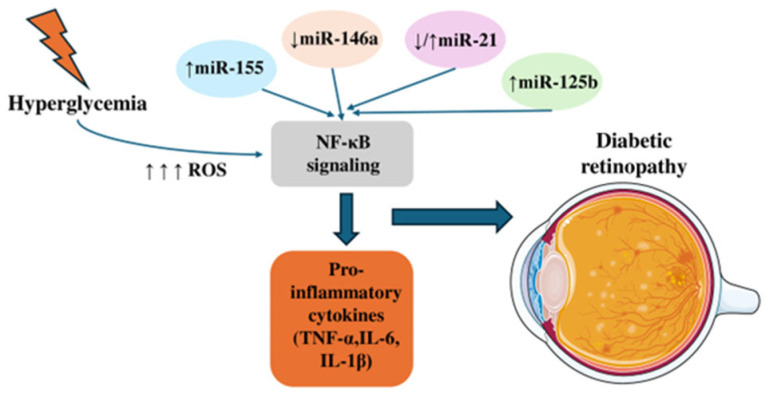
The effects of MicroRNAs on NF-κΒ signaling. Hyperglycemia increases reactive oxygen species (ROS), which activate NF-κB signaling. Altered microRNA expression—upregulation of miR-155 and miR-125b, downregulation of miR-146a, and variable changes in miR-21—further modulates NF-κB activity, leading to elevated pro-inflammatory cytokines (TNF-α, IL-6, IL-1β) and subsequent retinal alterations associated with diabetes.

MiR-29b, a member of the miR-29 family, is transcribed from miR-29b1 on chromosome 7q32.3 and miR-29b2 on chromosome 1q32.2, producing an identical mature miRNA [128]. MiR-29b has well-established neuroprotective and anti-apoptotic roles, particularly in neuronal cells subjected to neurotrophic deprivation, DNA damage, or endoplasmic reticulum stress [129]. Concomitantly, according to Qi et al. study, ethanol-induced miR-29b downregulation induces neuronal apoptosis via the SP1/RAX/PKR signaling cascade [130]. MiR-29b is differentially expressed in diabetes mellitus and has a neuroprotective role [131,132]. MiR-29b targets directly SP1, as miR-29b exerts its inhibitory effect on SP1 expression by targeting a seed sequence in the 3′ UTR region of the SP1 mRNA [133,134,135]. Zhang J et al. in their experimental study using STZ-induced DM mice and high glucose-treated retinal Müller cells (rMC-1) isolated from normal or STZ-induced mice, confirmed that hyperglycemia induced LncRNA MIAT upregulation, downregulates miR-29b expression, and thus SP1 expression is upregulated, inducing apoptosis of the retinal Müller cells in diabetic retinopathy [132]. Zeng K et al., in their experimental study using STZ-induced diabetic rats, high glucose treated retinal Muller cells and Resveratrol treatment, investigated the role miR-29b reduction has in SP1 expression and apoptosis of retinal cells in the inner nuclear layer (INL) in DR and further the role of miR-29b regulating Bax, Bcl-2 and SP1 in retinal Muller cells leading them to apoptosis [131]. In high glucose-treated retinal Müller cells, miR-29b expression is downregulated, causing SP1 upregulation, Caspase-3 increase, Bax protein expression increase, Bcl-2 protein expression decrease and thus promoting the Müller cells’ apoptosis [131]. Thus, under diabetic conditions, reduced miR-29b removes a critical inhibitory control over SP1, tipping the balance toward pro-apoptotic signaling in retinal Müller cells, which contributes to retinal neurodegeneration in DR (Table 2, Figure 2).

Early screening for specific miRNAs in diabetic patients without retinopathy may help detect precursors of retinal damage. Early treatment involving strict glycemic, blood pressure, and lipid control could contribute to preserving vision and enhancing the quality of life in individuals with diabetic retinopathy. Animal studies have demonstrated the potential of various miRNAs to show protective effects against diabetic retinopathy, although direct evidence of their therapeutic use in humans for preserving vision is still lacking. High glucose conditions lead to changes in microRNA expression within the diabetic retina, and specific miRNAs are found in both the peripheral serum and vitreous body. Dysregulation of miRNAs modulates the NF-κB pathway, leading to suppressed expression of antioxidant-related factors, and higher ROS production. Simultaneously, miRNAs interfere with vascular endothelial factor pathways, resulting in reduced vascular repair, enhanced endothelial cell migration, and disruption of the vascular barrier. Evidence from animal studies-most notably in diabetic mice-indicates that intravitreal miR-146 administration suppresses diabetes-induced NF-κB signaling, reduces microvascular leakage, and helps maintain retinal integrity [136]. Another preclinical study further supports this, showing that systemic injection of a miR-467 antagonist blocks hyperglycemia-induced vascular growth and angiogenesis in mice [137]. Although research in animal models indicates that miRNAs can improve diabetic symptoms, further investigations are essential to verify these effects and explore their therapeutic potential (Figure 2).

## 6. Conclusions

This review highlights the critical and multifaceted roles of microRNAs (miRNAs) in the pathogenesis of diabetic retinopathy (DR). Dysregulated miRNAs, including miR-155, miR-146a, miR-21, miR-126, miR-200b, miR-195, and miR-29b, profoundly influence key processes such as inflammation, endothelial dysfunction, neovascularization, fibrosis, and retinal cell apoptosis. Dysregulated expression of these miRNAs is implicated in microvascular damage, abnormal angiogenesis, and retinal neuronal loss. Elucidating the complex mechanisms mediated by miRNAs provides valuable insights for identifying novel biomarkers for early diabetic retinopathy (DR) diagnosis and designing targeted therapeutic interventions to slow disease progression.

## Figures and Tables

**Table 1:** Regulatory roles of key miRNAs in inflammatory and endothelial pathways of diabetic retinopathy.

miRNA	Regulation (↑/↓)	Main Pathways Mediated	Pathogenetic Mechanism in DR
miR-155 [16,17,18]	Upregulation	NF-κB, JAK/STAT, ROS, oxLDL-mediated inflammation	Pro-inflammatory M1 macrophage activation, cytokine production (IL-6, TNF-α), BRB breakdown, endothelial and pericyte apoptosis, vascular inflammation, and neointima formation
miR-146a [28,29,30]	Downregulation	NF-κB, TLR/IRAK1/TRAF6, ROS	Negative regulation of inflammation and oxidative stress, suppression of NF-κB and ROS, decreased TNF-α, IL-6, IL-12 production, and insulin resistance modulation
miR-21 [37,38,39]	Both (context-dependent)	NF-κB, NLRP, PPARα, ET-1, VCAM-1	Dual pro—and anti-inflammatory regulation, suppression of NF-κB, increased IL-10, endothelial dysfunction via ET-1/NO imbalance, VCAM-1 and MCP-1 mediated leukocyte adhesion
miR-125b [41,42]	Upregulation	MCP-1, EMT signaling	Anti-inflammatory effects via MCP-1 suppression, EMT induction in RPE cells, contributing to PDR
miR-126 [46,47]	Downregulation	PLK4, HIF-1α, TGFβ	Protection of endothelial integrity, inhibition of abnormal proliferation via PLK4 suppression, endothelial repair, reduction in TGFβ-induced apoptosis, and permeability

miR: microRNA; DR: Diabetic Retinopathy; NF-κB: Nuclear Factor kappa-light-chain-enhancer of activated B cells; JAK/STAT: Janus Kinase/Signal Transducer and Activator of Transcription; ROS: Reactive Oxygen Species; oxLDL: Oxidized Low-Density Lipoprotein; TLR: Toll-Like Receptor; IRAK1: Interleukin-1 Receptor-Associated Kinase 1; TRAF6: Tumor Necrosis Factor Receptor-Associated Factor 6; NLRP: NOD-, LRR- and Pyrin Domain-Containing Protein; PPARα: Peroxisome Proliferator-Activated Receptor Alpha; ET-1: Endothelin-1; VCAM-1: Vascular Cell Adhesion Molecule-1; MCP-1: Monocyte Chemoattractant Protein-1; EMT: Epithelial–Mesenchymal Transition; PDR: Proliferative Diabetic Retinopathy; RPE: Retinal Pigment Epithelium; PLK4: Polo-Like Kinase 4; HIF-1α: Hypoxia-Inducible Factor 1-alpha; TGFβ: Transforming Growth Factor Beta; IL-6/10/12: Interleukin-6/10/12; TNF-α: Tumor Necrosis Factor Alpha; BRB: Blood–Retinal Barrier; Upregulation (increased expression) of microRNAs means an increase in the amount or activity of that microRNA in a cell or tissue; Downregulation (decreased expression) of microRNAs means a decrease in the amount or activity of that microRNA.

**Table 2:** Key miRNAs mediating pathogenetic mechanisms driving diabetic retinopathy progression, including neovascularization, fibrosis, and apoptosis.

miRNA	Regulation (↑/↓)	Main Pathways Mediated	Pathogenetic Mechanism in DR
miR-126 [72,74,76,77]	Downregulation	VEGF, PI3K, MAPK (p38, ERK), MMP-9	Promotes angiogenesis via VEGF upregulation, p38/ERK activation, and endothelial cell cycle progression
miR-200b [68]	Downregulation	VEGF, Ets-1, MMP-1, VEGFR2, p300	Promotes angiogenesis via VEGF upregulation and Ets-1/p300-mediated transcriptional activation
miR-21 [85,91,109]	Upregulation	PTEN, PI3K/Akt, ERK, maspin	Promotes angiogenesis and fibrosis via PTEN suppression and PI3K/Akt/VEGF pathway activation
miR-155 [96]	Upregulation	SHIP1, PI3K/Akt	Promotes angiogenesis via SHIP1 downregulation and PI3K/Akt activation
miR-146a [33]	Downregulation	FN (Fibronectin)	Promotes fibrosis via FN upregulation in endothelial cells
miR-195 [119,121,122]	Upregulation	SIRT1, BAX, BCL-2	Promotes apoptosis via SIRT1 downregulation, increased BAX, and decreased BCL-2
miR-29b [129,132,133,135]	Downregulation	SP1, Bax, Bcl-2, Caspase-3	Promotes apoptosis in Müller cells via SP1 upregulation and BAX/BCL-2 imbalance

DR: Diabetic Retinopathy; miRNA: microRNA; VEGF: Vascular Endothelial Growth Factor; PI3K: Phosphoinositide 3-Kinase; MAPK: Mitogen-Activated Protein Kinase; ERK: Extracellular Signal-Regulated Kinase; MMP: Matrix Metalloproteinase; Ets-1: v-ets erythroblastosis virus E26 oncogene homolog 1; VEGFR2: Vascular Endothelial Growth Factor Receptor 2; PTEN: Phosphatase and Tensin Homolog; Akt: Protein Kinase B; SHIP1: SH2-containing Inositol 5′-Phosphatase 1; FN: Fibronectin; SIRT1: Silent Information Regulator Type 1; BAX: Bcl-2-Associated X Protein; BCL-2: B-cell Lymphoma 2; SP1: Specificity Protein 1; Upregulation (increased expression) of microRNAs means an increase in the amount or activity of that microRNA in a cell or tissue; Downregulation (decreased expression) of microRNAs means a decrease in the amount or activity of that microRNA.

## Data Availability

Not applicable.
